# Effects of seaweed on blood plasma immunoglobulin concentration, mucosal immunity, small intestine histomorphology, cecal microbial population, and volatile fatty acid profile in broiler chickens

**DOI:** 10.14202/vetworld.2025.508-518

**Published:** 2025-02-27

**Authors:** Mohammad Naeem Azizi, Teck Chwen Loh, Hooi Ling Foo, Wan Ibrahim Izuddin

**Affiliations:** 1 Department of Animal Science, Faculty of Agriculture, Universiti Putra Malaysia, Serdang 43400, Selangor, Malaysia; 2 Department of Pre-clinic, Faculty of Veterinary Science, Afghanistan National Agricultural Sciences and Technology University, Kandahar 3801, Afghanistan; 3 Institute of Tropical Agriculture and Food Security, Universiti Putra Malaysia, Serdang 43400, Selangor, Malaysia; 4 Department of Bioprocess Technology, Faculty of Biotechnology and Biomolecular Science, Universiti Putra Malaysia, Serdang 43400, Selangor, Malaysia; 5 Institute of Bioscience, Universiti Putra Malaysia, Serdang 43400, Selangor, Malaysia

**Keywords:** broiler chickens, cytokine expression, gut health, immunoglobulin, seaweed supplementation, sustainable poultry nutrition, volatile fatty acids

## Abstract

**Background and Aim:**

Seaweeds, particularly brown seaweed (BS) and green seaweed (GS), are rich in bioactive compounds that may enhance poultry health and productivity. This study evaluates the effects of dietary BS and GS on blood plasma immunoglobulin concentrations, mucosal immunity, small intestine histomorphology, cecal microbial populations, and volatile fatty acid (VFA) profiles in broiler chickens.

**Materials and Methods:**

A total of 504 one-day-old male broilers were randomly assigned to 12 dietary treatments: A negative control (basal diet), a positive control (basal diet + 100 mg/kg Vitamin E), and diets supplemented with BS and GS at 0.25%, 0.50%, 0.75%, 1.00%, and 1.25%. The study followed a completely randomized design, with data analyzed using a one-way analysis of variance and Duncan’s multiple range test (p < 0.05).

**Results:**

Broilers fed 0.75%, 1.00%, and 1.25% GS exhibited significantly higher (p < 0.05) blood plasma immunoglobulin A (IgA) and immunoglobulin G (IgG) concentrations. Dietary BS and GS inclusion upregulated messenger RNA expression of interleukin-6, interleukin-10, and interferon-gamma, indicating immunomodulatory effects. Jejunal villus height was significantly increased in birds fed 0.50%, 0.75%, and 1.25% BS during the starter period. Birds receiving 0.50% BS, 0.25% GS, and 0.50% GS exhibited higher cecal *Lactobacillus* counts, whereas 0.75% BS and GS significantly reduced *Escherichia coli* populations. Furthermore, higher total VFA and propionic acid concentrations were observed in birds supplemented with 1.00% and 1.25% GS, as well as 1.25% BS.

**Conclusion:**

The inclusion of GS (0.75%, 1.00%, and 1.25%) in broiler diets enhances immune response by increasing IgA and IgG levels. Both BS and GS positively modulate cytokine expression, intestinal morphology, and microbial balance, leading to improved gut health. The results suggest that BS and GS supplementation may serve as sustainable feed additives to enhance broiler performance while reducing reliance on synthetic supplements. Future studies should focus on identifying the bioactive compounds responsible for these effects and their broader implications for poultry production.

## INTRODUCTION

Seaweed is directly utilized in the human food industry, as well as in the chemical and pharmaceutical sectors, for the production of various chemicals and pharmaceutical compounds [1, 2]. Approximately 66.5% of brown seaweed (BS), 5% of green seaweed (GS), and 33% of red seaweed (RS) are consumed by humans in Asian countries, including Korea, Japan, and China [3]. On the other hand, seaweed has a long history of being used as livestock feed [4]. Feed supplements in broiler nutrition enhance feed effectiveness for cost-effective production and maintain and improve birds’ health. Natural feed supplements can produce antibiotic-free chicken meat [5].

The inclusion of 1.25% BS as well as 0.25%, 0.50%, 0.75%, and 1.00% GS in the diet of broiler chickens has been shown to enhance growth in broiler chickens [6]. It has been revealed that bioactive molecules contained in seaweed function as healthy prebiotics [7]. One of the most intriguing benefits of algae-based nutrition is its ability to positively impact the gastrointestinal microbiome of poultry, which plays a vital role in improving chicken digestive health and nutrient uptake [8]. They serve as beneficial bacteria substrates, alter microflora, and enhance the host immune system [9]. Seaweed sulfated polysaccharides exhibit potent immunostimulating properties because of the increased production of anti-inflammatory mediators [10]. Furthermore, seaweed is being investigated as a possible substitute for antibiotics in livestock diets [11, 12]. Seaweed’s dietary polysaccharides, including alginate, fucoidan, ulvan, and laminarin, have demonstrated potent antimicrobial activities [13, 14]. The antibacterial activity of seaweed is due to the inhibition of oxidative phosphorylation and link with bacteria’s cell wall content, increasing the permeability of the cytoplasmic membrane, which causes cell lysis [7, 15].

Hence, there is growing interest in using seaweed as a functional feed ingredient for broiler chickens [9]. This study aimed to study the effects of BS and GS on blood plasma immunoglobulin concentration, mucosal immunity, cecal microbial population, small intestine histomorphology, and volatile fatty acid (VFA) profile in broiler chickens.

## MATERIALS AND METHODS

### Ethical approval

The Institutional Animal Care and Use Committee of Universiti Putra Malaysia approved the guidelines used in this study (AUP-R093/2019). This article is part of a broader study that used identical experimental designs, diets, and animal husbandry. At the same time, previous parts of the research have already been made available in published form [5, 16].

### Study period and location

The feeding trial was conducted for a period of six weeks, from August to October, 2020 at the Department of Animal Science, Faculty of Agriculture, Universiti Putra Malaysia, Serdang 43400, Selangor, Malaysia.

### Experimental design

The BS and GS used in this study were mixtures of BS and GS and were used in whole form. Seaweed was provided by Promise Earth (M) Sdn. Bhd., a biotechnology company (Shah Alam 40200, Selangor, Malaysia).

A total of 504-day-old male broiler chickens (Cobb 500) were obtained from a local hatchery and reared for 42 days (from August 20 to October 1). The chickens were distributed into 12 dietary treatment groups (Each group consisted of six replicates, with each replicate containing seven birds): A basal diet, which was considered negative control (NC) with no seaweed inclusions; a basal diet + 100 mg/kg feed vitamin E as a positive control (PC); and basal diets + 0.25%, 0.50%, 0.75%, 1.00%, and 1.25% inclusion levels of GS and 0.25%, 0.50%, 0.75%, 1.00%, and 1.25% inclusion levels of BS. As previously mentioned, this publication is part of an extensive study in which the PC group was considered for examining factors linked to antioxidant-related parameters. The feed was formulated for the starter (Table 1) and finisher (Table 2) phases in accordance with the nutritional requirements of broilers [5, 16].

**Table 1:** Starter period diet.

Ingredients (%)^2^	Dietary treatments^1^											

	NC	PC	BS 0.25	BS 0.50	BS 0.75	BS 1	BS 1.25	GS 0.25	GS 0.50	GS 0.75	GS 1	GS 1.25
Corn	46.0	46.0	46.0	46.0	46.0	46.0	46.0	46.0	46.0	46.0	46.0	46.0
Soybean meal	40.0	40.0	39.8	39.5	39.3	39.0	38.8	39.8	39.5	39.3	39.0	38.8
Wheat pollard	5.0	5.0	5.0	5.0	5.0	5.0	5.0	5.0	5.0	5.0	5.0	5.0
Palm oil	4.0	4.0	4.0	4.0	4.0	4.0	4.0	4.0	4.0	4.0	4.0	4.0
L-Lysine	0.20	0.20	0.20	0.20	0.20	0.20	0.20	0.20	0.20	0.20	0.20	0.20
DL-Methionine	0.40	0.40	0.40	0.40	0.40	0.40	0.40	0.40	0.40	0.40	0.40	0.40
Dicalcium phosphate	2.60	2.60	2.60	2.60	2.60	2.60	2.60	2.60	2.60	2.60	2.60	2.60
Calcium carbonate	0.80	0.80	0.80	0.80	0.80	0.80	0.80	0.80	0.80	0.80	0.80	0.80
Choline chloride	0.20	0.20	0.20	0.20	0.20	0.20	0.20	0.20	0.20	0.20	0.20	0.20
Salt	0.30	0.30	0.30	0.30	0.30	0.30	0.30	0.30	0.30	0.30	0.30	0.30
Mineral mix	0.15	0.15	0.15	0.15	0.15	0.15	0.15	0.15	0.15	0.15	0.15	0.15
Vitamin mix	0.15	0.15	0.15	0.15	0.15	0.15	0.15	0.15	0.15	0.15	0.15	0.15
Antioxidants	0.10	0.10	0.10	0.10	0.10	0.10	0.10	0.10	0.10	0.10	0.10	0.10
Toxin binder	0.10	0.10	0.10	0.10	0.10	0.10	0.10	0.10	0.10	0.10	0.10	0.10
Seaweed	-	-	0.25	0.50	0.75	1.00	1.25	0.25	0.50	0.75	1.00	1.25
Vitamin E	-	0.01	-	-	-	-	-	-	-	-	-	-
Total	100	100	100	100	100	100	100	100	100	100	100	100
Calculated analysis												
Metabolizable energy (kcal/kg)	3040.16	3039.86	3041.02	3041.88	3042.74	3043.60	3044.46	3040.74	3041.31	3041.89	3042.48	3043.04
Protein	21.95	21.95	21.94	21.91	21.90	21.89	21.87	21.93	21.90	21.87	21.85	21.82
Fat	5.98	5.98	5.98	5.98	5.98	5.98	5.98	5.98	5.98	5.98	5.97	5.97
Fiber	4.34	4.34	4.33	4.31	4.31	4.29	4.28	4.32	4.31	4.30	4.29	4.28
Calcium	0.83	0.83	0.83	0.83	0.83	0.83	0.83	0.83	0.83	0.83	0.83	0.83
Total phosphorous	1.01	1.01	1.01	1.01	1.01	1.00	1.00	1.01	1.01	1.00	1.00	1.00
Available phosphorus	0.50	0.50	0.50	0.50	0.50	0.50	0.50	0.50	0.50	0.50	0.50	0.50

1 Dietary treatment: A negative control group (NC); a basal diet+Vitamin E (100 mg/kg of feed) as a positive control group (PC); and basal diets+0.25%, 0.50%, 0.75%, 1.00%, and 1.25% levels of brown seaweed (BS) and green seaweed (GS)

2 Azizi *et al*.[5,16]

**Table 2:** Finisher period diet.

Ingredients (%)^2^	Dietary treatments^1^											

	NC	PC	BS 0.25	BS 0.50	BS 0.75	BS 1	BS 1.25	GS 0.25	GS 0.50	GS 0.75	GS 1	GS 1.25
Corn	52.0	52.0	52.0	52.0	52.0	52.0	52.0	52.0	52.0	52.0	52.0	52.0
Soybean meal	32.0	32.0	31.8	31.5	31.3	31.0	30.8	31.8	31.5	31.3	31.0	30.8
Wheat pollard	6.0	6.0	6.0	6.0	6.0	6.0	6.0	6.0	6.0	6.0	6.0	6.0
Palm oil	5.10	5.10	5.10	5.10	5.10	5.10	5.10	5.10	5.10	5.10	5.10	5.10
L-Lysine	0.20	0.20	0.20	0.20	0.20	0.20	0.20	0.20	0.20	0.20	0.20	0.20
DL-Methionine	0.30	0.30	0.30	0.30	0.30	0.30	0.30	0.30	0.30	0.30	0.30	0.30
Dicalcium phosphate	2.40	2.40	2.40	2.40	2.40	2.40	2.40	2.40	2.40	2.40	2.40	2.40
Calcium carbonate	1.0	1.0	1.0	1.0	1.0	1.0	1.0	1.0	1.0	1.0	1.0	1.0
Choline chloride	0.20	0.20	0.20	0.20	0.20	0.20	0.20	0.20	0.20	0.20	0.20	0.20
Salt	0.30	0.30	0.30	0.30	0.30	0.30	0.30	0.30	0.30	0.30	0.30	0.30
Mineral mix	0.15	0.15	0.15	0.15	0.15	0.15	0.15	0.15	0.15	0.15	0.15	0.15
Vitamin mix	0.15	0.15	0.15	0.15	0.15	0.15	0.15	0.15	0.15	0.15	0.15	0.15
Antioxidants	0.10	0.10	0.10	0.10	0.10	0.10	0.10	0.10	0.10	0.10	0.10	0.10
Toxin binder	0.10	0.10	0.10	0.10	0.10	0.10	0.10	0.10	0.10	0.10	0.10	0.10
Seaweed	-	-	0.25	0.50	0.75	1.00	1.25	0.25	0.50	0.75	1.00	1.25
Vitamin E	-	0.01	-	-	-	-	-	-	-	-	-	-
Total	100	100	100	100	100	100	100	100	100	100	100	100
Calculated analysis												
Metabolizable energy (kcal/kg)	3149.82	3149.50	3150.68	3151.54	3152.40	3153.26	3154.12	3150.39	3150.97	3151.55	3152.13	3152.70
Protein	19.06	19.06	19.05	19.03	19.01	19.00	18.98	19.04	19.01	18.98	18.96	18.93
Fat	7.19	7.19	7.19	7.19	7.19	7.19	7.19	7.19	7.19	7.18	7.18	7.18
Fiber	4.00	4.00	3.99	3.98	3.97	3.96	3.95	3.99	3.98	3.97	3.96	3.94
Calcium	0.85	0.85	0.85	0.85	0.85	0.85	0.85	0.85	0.85	0.85	0.85	0.85
Total phosphorous	0.94	0.94	0.94	0.94	0.94	0.94	0.94	0.94	0.94	0.94	0.94	0.94
Available phosphorus	0.47	0.47	0.47	0.47	0.47	0.47	0.47	0.47	0.47	0.47	0.47	0.47

1 Dietary treatment: A negative control group (NC); a basal diet+vitamin E (100 mg/kg of feed) as a positive control group (PC); and basal diets+0.25%, 0.50%, 0.75%, 1.00%, and 1.25% levels of brown seaweed (BS) and green seaweed (GS).

2 Azizi *et al*.[5,16]

### Sample collection

Six birds were randomly selected from each group (one bird from a replicate) and slaughtered based on the Halal procedure outlined by the Department of Standard Malaysia, MS1500:2009, for sampling on days 21 and 42. Blood samples were collected during neck cutting, and plasma was harvested [5]. Intestinal sections were collected from the duodenum, jejunum, and ileum for histomorphological analysis. Cecum was collected to analyze the gut microbial population and VFA profile. A portion of the jejunum was collected for cytokine gene expression analysis.

### Chemical analysis

The concentrations of immunoglobulins were measured using commercial chicken immunoglobulin A (IgA), immunoglobulin M (IgM), and immunoglobulin G (IgG) enzyme-linked immunosorbent assay kits (QAYEE-BIO, Shanghai, China) [17].

To analyze cytokine gene expression, RNA extraction, RNA purity, and concentration evaluations, complementary DNA syntheses and real-time polymerase chain reaction (PCR) were performed as explained in the published part of the research [5, 16]. Glyceraldehyde-3-phosphate dehydrogenase was used as a housekeeping gene. Table 3 presents the target gene primer sequences.

**Table 3:** Target genes primer sequences.

Target gene	Primer sequence 5’→3’		bp	Accession No.
IL-1β	F-TGCTTCGTGCTGGAGTCACCC	R-GGCCGGTACAGCGCAATGTT	98	XM_015297469.2
IL-6	F-GCTCGCCGGCTTCGA	R-GGTAGGTCTGAAAGGCGAACAG	71	NM_204628.1
IL-8	F-GGCTTGCTAGGGGAAATGA	R-AGCTGACTCTGACTAGGAAACTGT	200	NM_205498.1
IL-10	F-TAACATCCAACTGCTCAGCTC	R-TGATGACTGGTGCTGGTCTG	135	NM_001004414.2
IFN-γ	F-GAGCCATCACCAAGAAGATGA	R-TAGGTCCACCGTCAGCTACA	177	NM_205149.1
TNF-α	F-GCTGTTCTATGACCGCCCAGTT	R-AACAACCAGCTATGCACCCCA	140	XM_040647309.1
GAPDH	F-CTGGCAAAGTCCAAGTGGTG	R-AGCACCACCCTTCAGATGAG	275	NM_204305.1

F=Forward, R=Reverse. bp (base pair)=Product size. IL-1β=Interleukin-1β, IL-6=Interleukin 6, IL-8=Interleukin 8, IL-10=Interleukin 10, IFN-γ=Interferon gamma, TNF-α=Tumor necrosis factor alpha, GAPDH=Glyceraldehyde-3-phosphate dehydrogenase

As explained previously by Danladi *et al*. [17], the histomorphology of the small intestine was determined by the villi height and crypt depth. To determine the microbial population of cecal content, DNA was extracted from the cecal contents using a NucleoSpin^®^ DNA Stool kit (MACHEREY-NAGEL, Allentown, USA) based on the manufacturer’s instructions. Ultraviolet-visible spectroscopy (absorbance 260/280) was used to determine the concentration and purity of DNA (Multiskan, Thermo Scientific, USA). Real-time PCR was performed using a LightCycler^®^ 480 quantitative PCR (qPCR) system (Roche Molecular Systems, USA). A qPCR master mix (20 μL) was prepared using a CAPITALTM qPCR Green Mix, 4×, based on the manufacturer’s instructions (Biotechrabbit, Hennigsdorf, Germany).

The qPCR was conducted using the LightCycler^®^ 480 qPCR system (Roche Molecular Systems, Inc.). Cecum microbes were quantified based on target microbe amplification, as described by Humam *et al*. [18]. The sequences of the targeted microbes are shown in Table 4 [19–21].

**Table 4:** The primer sequences of cecal-targeted microbes.

Target microbes	Primer sequence 5′→3′		bp	References
Total bacteria	F-CGGCAACGAGCGCAACCC	R-CCATTGTAGCACGTGTGTAGCC	145	[19]
*Enterococcus*	F-CCCTTATTGTTAGTTGCCATCATT	R-ACTCGTTGTACTTCCCATTGT	144	[19]
Enterobacteriaceae	F-CATTGACGTTACCCGCAGAAGAAGC	R-CTCTACGAGACTCAAGCTTGC	195	[20]
*Lactobacillus*	F-CATCCAGTGCAAACCTAAGAG	R-GATCCGCTTGCCTTCGCA	341	[20]
*Escherichia coli*	F-GTGTGATATCTACCCGCTTCGC	R-AGAACGCTTTGTGGTTAATCAGGA	82	[20]
*Bifidobacterium*	F-GGGTGGTAATGCCGGATG	R-TAAGCCATGGACTTTCACACC	278	[21]

F=Forward, R=Reverse. bp (base pair)=Product size

The VFA concentration in the cecum was determined using a gas chromatography system (Agilent 6890N Series, Agilent Technologies, USA) based on the method explained by Thanh *et al*. [22].

### Statistical analysis

All data were analyzed using the General Linear Model procedure in the Statistical Analysis System software (SAS 9.4, SAS Institute Inc., Cary, NC, USA). A one-way analysis of variance was performed to determine the effects of dietary treatments on measured parameters. Duncan’s multiple range test was used for *post hoc* comparisons to identify significant differences among treatment means at p < 0.05. In addition, orthogonal polynomial contrast analysis was conducted to assess the linear and quadratic effects of increasing dietary BS and GS inclusion levels. Results are presented as mean ± standard error of the mean, ensuring statistical robustness in the interpretation of findings.

## RESULTS AND DISCUSSION

### Immunoglobulin concentration

The effects of BS and GS on broiler plasma IgA, IgM, and IgG concentrations are presented in Table 5. Various levels of GS (0.75%, 1.00%, and 1.25%) improved (p < 0.05) blood plasma IgA and IgG concentrations. Nevertheless, no significant difference (p > 0.05) was determined for the plasma immunoglobulin concentration in the BS groups compared to the control groups.

**Table 5:** Effects of seaweed on plasma IgA, IgM, and IgG concentration.

Parameters^1^	Dietary treatments^2^												SEM^3^	p-values	Contrast p-values	
	
	NC	PC	BS 0.25	BS 0.50	BS 0.75	BS 1	BS 1.25	GS 0.25	GS 0.50	GS 0.75	GS 1	GS 1.25			Linear	Quadratic
IgA (ng/mL)	135.8^d^	144.71^cd^	140.69^cd^	146.72^cd^	151.3^cd^	169.7^bcd^	161.7^bcd^	165.7^bcd^	166.8^bcd^	172.3^bc^	189.54^ab^	213.39^a^	10.27	0.0153	0.3143	0.6788
IgM (μg/mL)	59.67	61.46	59.87	63.69	63.54	68.01	67.71	69.64	63.84	72.17	74.55	71.28	4.70	0.3897	0.8010	0.4217
IgG (ng/mL)	201.9^bc^	195.36^c^	195.95^c^	197.74^c^	217.9^abc^	244.7^abc^	239.4^abc^	241.8^abc^	224.5^abc^	275.12^ab^	293.57^a^	290^a^	22.82	0.0001	0.7878	0.6114

^a,b,c^A significant difference (p < 0.05) is shown by means in the same row with distinct superscripts.

1 Parameters: IgA=Immunoglobulin A, IgM=Immunoglobulin M, IgG=Immunoglobulin G.

2 Dietary treatment: A negative control group (NC); a basal diet + vitamin E (100 mg/kg of feed) as a positive control group (PC); and basal diets + 0.25%, 0.50%, 0.75%, 1.00%, and 1.25% levels of brown seaweed (BS) and green seaweed (GS).

3 SEM=Standard error of the mean

IgA interacts with specific receptors and immunological mediators to mediate various protective functions [17, 23]. As the initial immunological response to antigens, IgM controls the immune response and accelerates IgG synthesis [24]. IgG binds to the antigen, making it more visible for phagocytic cells to remove them or their toxic products from the body [25].

In an earlier study by Choi *et al*. [26], an increase in IgA production was reported for broiler chickens fed 0.50% BS by-product fermented by *Bacillus subtilis* and *Aspergillus oryzae*. In another study by Bussy *et al*. [27], 16 g/day of seaweed extract increased anti-*Bordetella* IgG levels in sow blood and colostrum. The sulfated polysaccharides isolated from GS stimulated macrophages, which produced pro- and anti-inflammatory cytokines, indicating their potential as immunostimulants [28]. The improvement in immunoglobulin concentration in this study might be due to the ulvan polysaccharide of GS. Previous research by Venkatesan *et al*. [29] indicated that ulvan exhibits immunomodulating activity. Incubation of macrophages with ulvan extract was established to increase nitric oxide production, which usually occurs during host defense against antigens [29].

### Cytokine gene expression

The result (Table 6) showed that birds fed 0.50%, 0.75% BS, and 1.25% GS significantly upregulated interleukin-6 (IL-6) gene messenger RNA (mRNA) expression compared with the NC group. Furthermore, birds fed 0.75% or 1.25% GS exhibited significantly higher interleukin-10 (IL-10) mRNA expression than the NC group. Meanwhile, 0.50% BS, 0.75%, 1.00% GS, and 1.25% GS increased (p < 0.05) the interferon-gamma (IFN-γ) gene mRNA expression compared to the NC group. No significant difference (p > 0.05) was observed in the mRNA expression for interleukin-1 (IL-1β) and tumor necrosis factor-alpha (TNF-α) among dietary treatment groups.

**Table 6:** Effects of seaweed on cytokine messenger RNA (mRNA) expression.

Parameters^1^ (mRNA fold change)	Dietary treatments^2^												SEM^3^	p-values	Contrast p-values^4^	
	
	NC	PC	BS 0.25	BS 0.50	BS 0.75	BS 1	BS 1.25	GS 0.25	GS 0.50	GS 0.75	GS 1	GS 1.25			Linear	Quadratic
IL-1β	1	0.990	0.867	1.164	1.120	0.996	1.029	0.980	1.185	1.358	1.159	1.227	0.044	0.5996	0.3844	0.4710
IL-6	1^c^	1.213^abc^	1.077^bc^	1.757^a^	1.714^ab^	1.219^abc^	0.870^c^	1.342^abc^	1.305^abc^	1.438^abc^	1.112^bc^	1.754^a^	0.068	0.0354	0.6471	0.1362
IL-8	1	1.026	1.014	1.136	1.026	0.993	1.045	0.937	1.359	1.144	1.299	1.260	0.044	0.8470	0.5298	0.8565
IL-10	1^bc^	1.091^abc^	1.235^abc^	1.028^bc^	1.132^abc^	1.080^bc^	0.946^c^	0.924^c^	1.085^bc^	1.397^a^	1.312^abc^	1.476^a^	0.043	0.0254	0.0032	0.0261
IFN-γ	1^c^	1.119^bc^	1.084^c^	1.751^ab^	1.171^bc^	1.246^bc^	1.147^bc^	1.049^c^	1.236^bc^	1.916^a^	1.890^a^	1.911^a^	0.077	0.0092	0.0065	0.0032
TNF-α	1	1.124	1.099	1.279	0.783	1.076	1.124	1.040	1.070	0.888	0.771	1.015	0.047	0.5967	0.2325	0.6251

^a,b,c^A significant difference (p < 0.05) is shown by means in the same row with distinct superscripts.

1 Parameters: IL-1β=Interleukin-1β, IL-6=Interleukin 6, IL-8=Interleukin 8, IL-10=Interleukin 10, INF-γ=Interferon-gamma, TNF-α=Tumor necrosis factor-alpha.

2 Dietary treatment: A negative control group (NC); a basal diet + vitamin E (100 mg/kg of feed) as a positive control group (PC); and basal diets + 0.25%, 0.50%, 0.75%, 1.00%, and 1.25% levels of brown seaweed (BS) and green seaweed (GS).

3 SEM=Standard error of means

In a previous study by Yan *et al*. [30], the effects of 0.04% sodium alginate oligosaccharides from BS on the mucosal immune responses of *Salmonella*-challenged broiler chickens showed that the IL-10 expression was significantly upregulated. Furthermore, the 1.00% GS level positively affected the IL-6 and 0.80% of seaweed on the IFN-γ in laying chickens [31]. Similarly, strong immunomodulatory activities of GS-sulfated polysaccharides were reported in an *in vitro* study because of the increased production of IL-6 and TNF-α [10]. Seaweed sulfated polysaccharides are potent immunostimulators that trigger immune cell activity and enhance the immunological response [32].

### Small intestine histomorphology

Table 7 illustrates the impact of seaweed levels on broiler villus height, crypt depth, and the villus height: crypt depth ratio in the small intestine. Compared with the NC group, the duodenal villus height and villus height: crypt depth ratios were not significantly different between the seaweed-supplemented and non-supplemented groups in the starter and finisher periods. In contrast, the crypt depth of the duodenum villi was linearly and quadratically increased (p < 0.05) in the 0.50% BS and 1.25% GS supplementation groups compared with the NC group in the finisher period. In the starter period, birds fed 0.50, 0.75, or 1.25% BS had significantly higher jejunal villus heights. Furthermore, jejunum’s villus height: crypt depth ratio was significantly higher in birds fed 0.75% BS than in the NC group during the starter period. In contrast to the NC, different levels of BS and GS had no significant effects on the villus height, crypt depth, and villus height: crypt depth ratio of the jejunum in the finisher period. A linear improvement (p < 0.05) was observed for 1.00% and 1.25% GS in the crypt depth of the ileum during the finisher period. However, no effects (p > 0.05) were observed on villus height, crypt depth, and the villus height: crypt depth ratio in the ileum among other treatment groups in the starter and finisher periods of chickens.

**Table 7:** Effects of seaweed intestinal histomorphology.

Parameters (μM)	Dietary treatments^1^												SEM^2^	p-values	Contrast P-values	
	
	NC	PC	BS 0.25	BS 0.50	BS 0.75	BS 1	BS 1.25	GS 0.25	GS 0.50	GS 0.75	GS 1	GS 1.25			Linear	Quadratic
0-3 weeks (Starter period)																
Villus height																
Duodenum	989.59	1009.66	1017.60	1043.53	1012.55	1049.88	990.67	1048.83	1011.86	1011.02	985.16	1035.16	20.99	0.5721	0.1763	0.1324
Jejunum	543.7^c^	608.5^ab^	542.8^c^	606.9^ab^	605.16^ab^	556.3^abc^	611.98^a^	570.3^abc^	557.9^abc^	597.12^abc^	549.61^bc^	574.98^abc^	17.07	0.0383	0.3165	0.0159
Ileum	432.79	449.79	387.22	409.21	391.51	435.24	405.15	457.80	408.51	401.43	407.91	389.49	21.42	0.7153	0.4150	0.3602
Crypt depth																
Duodenum	157.6	145.4	175.52	156.5	146.1	146.0	153.5	182.7	165.0	156.6	142.1	169.5	8.02	0.1180	0.3010	0.7033
Jejunum	129.1^abc^	123.49^bc^	115.62^c^	129.1^abc^	117.87^c^	122.76^bc^	138.42^a^	135.2^ab^	125.5^abc^	137.52^a^	126.9^abc^	133.76^ab^	4.13	0.0052	0.7996	0.5225
Ileum	121.48	110.62	103.20	115.81	114.43	115.29	112.39	112.18	113.23	127.76	119.65	123.84	6.60	0.7128	0.1028	0.0986
Villus height and crypt depth ratio																
Duodenum	5.74	6.98	5.82	6.79	7.05	7.28	6.51	5.82	6.41	6.67	7.15	6.13	0.38	0.1742	0.7418	0.1109
Jejunum	4.24^c^	4.94^ab^	4.7^abc^	4.74^abc^	5.13^a^	4.55^abc^	4.44^bc^	4.23^c^	4.49^bc^	4.36^bc^	4.37^bc^	4.32^bc^	0.19	0.0330	0.3775	0.0290
Ileum	3.63	3.63	3.63	3.63	3.63	3.63	3.63	3.63	3.63	3.63	3.63	3.63	0.22	0.3154	0.3595	0.5183
4-6 weeks (Finisher period)																
Villus height																
Duodenum	1237.37	1210.43	1185.79	1255.77	1282.71	1240.39	1290.44	1246.12	1264.88	1252.00	1225.51	1275.00	23.92	0.1677	0.1940	0.9091
Jejunum	822.4^abc^	790.34	849.56^ab^	785.74^bc^	746.05^c^	807.1^abc^	773.49^bc^	784.28^bc^	825.3^abc^	840.04^ab^	837.29^ab^	871.30^a^	23.65	0.0474	0.0185	0.2571
Ileum	471.55	505.25	509.61	463.14	501.31	487.10	451.07	453.14	466.65	491.21	436.36	449.13	17.19	0.0618	0.6791	0.4449
Crypt depth																
Duodenum	141.4^cde^	163.0^abc^	155.2^abcd^	169.94^a^	143.4^cde^	150.5^abcd^	125.96^e^	142.4^cde^	146.4^bcde^	155.3^abcd^	138.92^de^	168.97^ab^	6.97	<.0001	0.0489	0.0309
Jejunum	137.8^ab^	135.06	127.71^bc^	119.04^b^	116.79^b^	138.25^ab^	123.48^ab^	121.7^ab^	120.99^b^	125.14^ab^	142.99^a^	133.35^ab^	6.78	0.2591	0.0315	0.3199
Ileum	107.5^bc^	121.49	118.0^abc^	113.3^abc^	107.47^bc^	101.19^c^	104.80^c^	106.43^c^	108.98^bc^	124.60^a^	122.35^a^	103.03^c^	5.77	0.1347	0.0110	0.3683
Villus height and crypt depth ratio																
Duodenum	8.89^abc^	7.48^c^	7.7^bc^	7.46^c^	9.02^abc^	9.68^ab^	10.28^a^	8.8^abc^	8.65^abc^	8.17^bc^	8.87^abc^	7.61^bc^	0.49	0.0021	0.0292	0.1421
Jejunum	6.06	6.01	6.71	6.64	6.44	5.92	6.36	6.53	6.89	6.75	6.00	6.60	0.40	0.8371	0.5055	0.8571
Ileum	4.48	4.17	4.34	4.18	4.71	4.83	4.35	4.33	4.37	3.91	3.54	4.38	0.28	0.2837	0.0713	0.7145

^a,b,c^A significant difference (p < 0.05) is shown by means in the same row with distinct superscripts.

1 Dietary treatment: A negative control group (NC); a basal diet + vitamin E (100 mg/kg of feed) as a positive control group (PC); and basal diets + 0.25%, 0.50%, 0.75%, 1.00%, and 1.25% levels of brown seaweed (BS) and green seaweed (GS).

2 SEM=Standard error of means

The density and size of the small intestine villi are directly related to the birds’ nutrient absorption ability [33–35]. Previous studies by Oretomiloye and Adewole [36], Sweeney *et al*. [37], and Sweeney *et al*. [38] have demonstrated that seaweed can improve broiler intestinal morphology by increasing the villi height. In a recent study by Oretomiloye and Adewole [36], dietary supplementation with BS improved intestinal morphology by enhancing the villus height and villus height: crypt depth ratio among heat-stressed birds. It was reported that 1000 parts per million (ppm) of BS extract significantly increased the villus height of broiler chickens, whereas 1000 ppm supplementation did not significantly affect birds’ small intestine morphology [37]. Laminarin and fucoidan extracts (250 ppm laminarin and 250 ppm laminarin + 80 ppm fucoidan) supplementation in broilers’ diets increased the height and width of villi [38]. Nevertheless, Mohammadigheisar *et al*. [39] have indicated that seaweed does not affect the morphology of the small intestine. A blend of brown, green, and red seaweed at 5, 10, and 20 g/kg in broiler feed did not affect jejunal histomorphology [39]. However, the effect of seaweed on intestinal histomorphology may depend on the seaweed’s chemical composition.

### Cecal microbial population

The cecal *Lactobacillus* populations of the birds fed 0.50% BS, 0.25, and 0.50% GS increased (p < 0.05) compared with the NC group (Table 8). On the other hand, 0.75% BS and GS significantly decreased the cecum content of *Escherichia* *coli* compared with the NC group. According to the literature, seaweed supplementation enhances animal intestinal microflora by increasing beneficial bacteria and reducing pathogens [36, 40, 41]. For instance, a 1.00% BS diet in pigs improved the intestinal microflora by increasing *Lactobacillus* counts and decreasing *E. coli* and *Shigella* counts [40]. In addition, Laminarin has been proven to possess prebiotic properties by improving the abundance of *Lactobacillus* [41]. Similarly, dietary supplementation with BS enhanced *Lactobacillus* in heat-stressed bird cecum [36]. Seaweed-sulfated and carboxylated polysaccharides such as ulvan, fucoidan, and alginates act as prebiotics and enhance the growth of beneficial bacteria by improving the gut [7, 14, 32]. The nutritional components of seaweed may optimize the intestinal micro-ecological environment and accelerate the replication of *Lactobacillus* colonies [17, 32, 42]. In the present study, no significant differences were observed in the total bacteria, *Enterobacteriaceae*, *Bifidobacterium*, and Enterococcus populations of cecum content among the dietary treatment groups.

**Table 8:** Effects of seaweed on cecum microbial populations (log_10_ CFU/g).

Parameters	Dietary treatments^1^												SEM^2^	p-values	Contrast p-values	
	
	NC	PC	BS 0.25	BS 0.50	BS 0.75	BS 1	BS 1.25	GS 0.25	GS 0.50	GS 0.75	GS 1	GS 1.25			Linear	Quadratic
Total bacteria	9.401	9.341	9.451	9.019	10.411	9.645	9.620	9.729	9.815	9.750	9.904	9.553	0.093	0.4516	0.7722	0.8031
*Enterobacteriaceae*	5.937	5.733	5.809	6.215	5.085	5.704	5.399	5.822	5.784	6.143	5.892	5.751	0.069	0.1262	0.1003	0.6510
*Lactobacillus*	6.872^bc^	7.734^abc^	7.598^abc^	8.195^a^	7.145^abc^	6.687^c^	7.389^abc^	8.271^a^	8.189^a^	6.887^bc^	7.620^abc^	7.904^ab^	0.120	0.0309	0.1701	0.1882
*Bifidobacterium*	8.758	8.982	9.045	8.937	8.916	8.912	8.779	9.243	8.954	8.905	8.811	9.169	0.043	0.3598	0.4536	0.5276
*Enterococcus*	7.547	8.138	7.715	8.019	7.598	7.492	7.853	7.722	7.878	7.401	7.918	7.399	0.060	0.1831	0.2750	0.8584
*Escherichia coli*	6.846^a^	6.430^abc^	6.596^abc^	6.614^ab^	6.047^bc^	6.532^abc^	6.878^a^	6.829^a^	7.067^a^	5.946^c^	6.488^abc^	6.397^abc^	0.073	0.0205	0.0135	0.0013

^a,b,c^A significant difference (p < 0.05) is shown by means in the same row with distinct superscripts.

1 Dietary treatment: A negative control group (NC); a basal diet + vitamin E (100 mg/kg of feed) as a positive control group (PC); and basal diets + 0.25%, 0.50%, 0.75%, 1.00%, and 1.25% levels of brown seaweed (BS) and green seaweed (GS).

2 SEM=Standard error of means, CFU=Colony-forming units

### Cecal VFA concentrations

The results of this study revealed that acetic acid was the major VFA in broiler cecum, followed by butyric and propionic acids (Table 9). Birds fed the 1.25% BS-, 1.00%, and 1.25% GS-based diets had higher (p < 0.05) propionic acid content than those fed the NC group. In addition, the total VFAs were higher (p < 0.05) in birds fed 1.00% and 1.25% BS and 0.75%, 1.00%, and 1.25% GS than in the control groups.

**Table 9:** Effects of brown and green seaweed on cecum volatile fatty acids (mM).

VFA^1^	Dietary treatments^2^												SEM^3^	p-values	Contrast p-values^4^	
	
	NC	PC	BS 0.25	BS 0.50	BS 0.75	BS 1	BS 1.25	GS 0.25	GS 0.50	GS 0.75	GS 1	GS 1.25			Linear	Quadratic
Acetic acid	52.07	48.61	49.18	56.37	53.85	71.65	59.05	61.42	61.02	64.49	67.52	64.01	1.77	0.2589	0.4165	0.5104
Propionic acid	7.40^d^	8.15^cd^	7.70^cd^	10.41^abcd^	9.54^cd^	9.99^bcd^	12.5^abc^	10.9^abcd^	10.6^abcd^	11.53^abcd^	15.29^a^	14.77^ab^	0.53	0.0269	0.7283	0.0821
Isobutyric acid	1.63	1.28	1.35	0.85	1.61	1.39	1.57	1.68	1.28	1.25	1.18	1.03	0.08	0.3190	0.3508	0.1420
Butyric acid	26.24	23.63	35.13	29.59	26.06	36.78	41.44	35.3	35.53	39.26	41.29	40.22	1.53	0.2339	0.7327	0.3560
Isovaleric acid	2.16	1.62	2.01	1.32	2.11	1.89	2.32	2.24	1.54	1.68	1.45	1.20	1.11	0.3758	0.2927	0.3018
Valeric acid	2.18	2.27	2.87	2.50	2.34	2.35	2.43	2.42	3.16	3.44	2.31	3.55	0.17	0.8884	0.6714	0.8062
Total VFA	91.68^de^	85.56^e^	98.24^bcde^	101.1^bcde^	95.53^cde^	124.05^ab^	119.3^abc^	113.9^abcd^	113.1^abcd^	121.64^abc^	129.05^a^	124.76^ab^	3.06	0.0156	0.4375	0.1878

^a,b,c^A significant difference (p < 0.05) is shown by means in the same row with distinct superscripts.

1 VFA=Volatile fatty acids.

2 Treatments: A negative control group (NC); a basal diet + vitamin E (100 mg/kg of feed) as a positive control group (PC); and basal diets + 0.25%, 0.50%, 0.75%, 1.00%, and 1.25% levels of brown seaweed (BS) and green seaweed (GS).

3 SEM=Standard error of means

The results of the current study on the cecum microbial population proved that various BS and GS groups have probiotic effects on broiler chickens. Hence, one of the foremost reasons for the higher propionic acid and total VFA contents may be the increase in gut-beneficial bacteria, such as *Lactobacillus*, in birds fed seaweed-supplemented feed [43]. In addition, seaweed polysaccharides such as laminarins and fucoidan have a complex structure, and they are dietary fibers that can modulate intestinal metabolism through intestinal pH, mucus composition, and short-chain fatty acid production [36, 44, 45].

## CONCLUSION

This study demonstrates that dietary supplementation with GS and BS positively influences immune function, gut microbiota, and intestinal morphology in broiler chickens. Notably, supplementation with 0.75%, 1.00%, and 1.25% GS significantly increased blood plasma IgA and IgG concentrations, indicating enhanced immune response. Both BS and GS upregulated the expression of key immune-related genes, including IL-6, IL-10, and IFN-γ, suggesting immunomodulatory effects. Improved jejunal villus height was observed in birds fed 0.50%, 0.75%, and 1.25% BS during the starter period, which may enhance nutrient absorption. In addition, 0.50% BS, 0.25% GS, and 0.50% GS increased *Lactobacillus* populations in the cecum, while 0.75% BS and GS effectively reduced *E. coli* expression level indicating a probiotic-like effect. Higher total VFA concentrations, particularly propionic acid, were observed in birds receiving 1.00% and 1.25% GS and 1.25% BS, further supporting the gut health benefits of seaweed supplementation.

The study provides a comprehensive assessment of multiple physiological and microbiological parameters, offering a holistic understanding of seaweed supplementation in poultry. By highlighting the immunomodulatory potential of GS and BS, it contributes to the growing interest in natural feed additives as sustainable alternatives to synthetic growth promoters and antibiotics. However, the study has certain limitations, including the short-term experimental duration, which does not account for long-term performance and health effects. Furthermore, while the study explores different inclusion levels, the optimal dosage for maximizing economic viability and biological efficacy requires further refinement. In addition, the focus was limited to BS and GS, whereas other seaweed species or their extracts might exhibit different or more pronounced effects.

Future research should aim to identify and characterize the bioactive compounds responsible for these observed benefits, investigate the molecular mechanisms underlying their effects on gut microbiota and immune modulation, and evaluate their application in other livestock species. A long-term assessment of seaweed supplementation in commercial broiler production, including economic feasibility and sustainability analysis, would further strengthen its potential as a viable feed additive. Overall, the findings highlight the potential of BS and GS to enhance broiler health and performance while reducing reliance on synthetic additives, supporting the broader goal of sustainable and antibiotic-free poultry production.

## AUTHORS’ CONTRIBUTIONS

MNA, TCL and HLF: Conceptualization. TCL and HLF: Supervision. MNA and WII: Chemical analysis. MNA and TCL: Statistical analysis. MNA: Manuscript first draft preparation. MNA, TCL, HLF, and WII: Reviewed and edited the manuscript. All authors have read and agreed to the final version of the manuscript.
